# Perceived effectiveness of endometriosis therapies on fatigue: an international survey

**DOI:** 10.1530/RAF-25-0010

**Published:** 2025-05-15

**Authors:** Kevin K W Kuan, Florian Heinzl, Andrew W Horne, Lucy H R Whitaker, Jana Heine, Christine Bekos

**Affiliations:** ^1^ Edinburgh Medical School, University of Edinburgh, Edinburgh, UK; ^2^ Department of Obstetrics and Gynecology, Medical University of Vienna, Vienna, Austria; ^3^ Centre for Reproductive Health, Institute of Inflammation and Repair, University of Edinburgh, Edinburgh, UK

**Keywords:** endometriosis therapy, fatigue, medical treatments, surgical interventions, comprehensive management strategies, international survey

## Abstract

**Graphical abstract:**

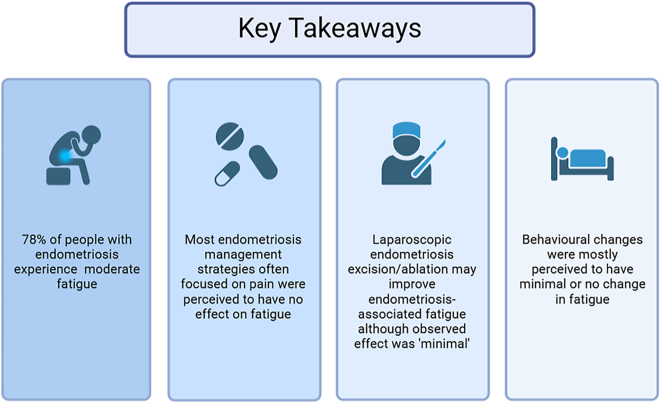

**Abstract:**

Current endometriosis treatments primarily focus on pain management, despite many patients also experiencing fatigue, which significantly impacts their quality of life. This study aimed to evaluate the perceived effectiveness of endometriosis treatments in managing fatigue. An international anonymous survey was conducted using the Qualtrics platform, with participants (aged 16 years and over) and a self-reported diagnosis of endometriosis. The survey collected demographic information, the brief fatigue inventory, and perceived impact of treatments on fatigue over the past 5 years. Ethical approval was granted by the Edinburgh Medical School Research Ethics Committee. Data analysis was performed using R, with results presented as medians and interquartile ranges. From 12 April to 25 May 2023, 2,907 responses were collected. Our results showed that fatigue was significantly worsened during menstruation (median: −2, IQR: −3 to −1) and slightly worsened during ovulation (median: −1, IQR: −2 to 0). Analysis revealed limited associations between common medical treatments, such as analgesics or hormonal therapy, and improvements in fatigue symptoms. Use of gonadotrophin-releasing hormone (GnRH) agonists was linked to a worsened fatigue, reported by 54% users. Surgical interventions and changes in rest patterns showed minimal improvement, while other behavioural modifications showed little to no effect. These findings suggest that current endometriosis treatments are largely ineffective in addressing fatigue. Limitations of this study include recall bias and confounding factors, which may influence perceived effectiveness of endometriosis therapies on fatigue. This underscores the need for more comprehensive management strategies to better support patients experiencing endometriosis-associated fatigue.

**Lay summary:**

Endometriosis is a common chronic pain condition affecting 180 million women worldwide. Many women with endometriosis also report that fatigue significantly impacts their quality of life. Despite this, fatigue management has been largely neglected, and there are limited studies that have evaluated the benefit of current endometriosis treatments on fatigue. This international survey aimed to evaluate the perceived effectiveness of various therapies for endometriosis on fatigue, including pain medication, hormonal medication, surgery and behavioural changes. Pain medication and most hormonal therapies provided limited relief. However, gonadotrophin-releasing hormone (GnRH) agonists worsened fatigue in more than half of the users. These results suggest that existing endometriosis treatments are largely ineffective in addressing fatigue, highlighting the need for improved strategies to address this symptom to enhance quality of life for women with endometriosis.

## Introduction

Endometriosis is a chronic neuroinflammatory disease affecting 10% of women and people assigned female at birth. Common symptoms include chronic pelvic pain, dysmenorrhoea and dyspareunia (Horne & Missmer 2022). Fatigue is another common symptom of endometriosis, affecting up to 87% of women, described as a persistent feeling of weariness, weakness or depleted energy (Pigeon *et al.* 2003, Ramin-Wright *et al.* 2018, Soliman *et al.* 2021). It can significantly impair all aspects of quality of life, including work, social activities, relationships, sexual functioning and mental health, often manifesting as increased depression and anxiety (DiBenedetti *et al.* 2020, Mundo-López *et al.* 2020, Soliman *et al.* 2021).

Traditionally, endometriosis management focused on reducing pain symptoms, and options include analgesia, hormonal therapy, surgery and complementary therapy (Becker *et al.* 2022). Various meta-analyses have identified certain hormonal strategies (e.g. combined hormonal contraceptive pill, progesterone or gonadotrophin-releasing hormone (GnRH) analogue) and surgical interventions to provide pain relief, although responses from patients can also be very heterogeneous (Chaichian *et al.* 2017, Mira *et al.* 2018, Singh *et al.* 2020). There has been growing interest in other forms of lifestyle management strategies, such as low-inflammatory diets and changes in sleep pattern, although research outcomes have often been centred around pain (Chaichian *et al.* 2017, Barnard *et al.* 2023).

The model for endometriosis management has now shifted towards a more holistic approach, ensuring biopsychosocial symptoms associated with endometriosis, including fatigue, are also addressed (Mick *et al.* 2024). While fatigue symptoms can be correlated with chronic pain, some patients may find symptoms of fatigue more bothersome than pain (DiBenedetti *et al.* 2020). Despite the high prevalence of endometriosis-associated fatigue and significant impact on a person’s quality of life, there is limited evidence regarding its management within the current literature (Ramin-Wright *et al.* 2018).

This study aims to assess the perceived effectiveness of existing endometriosis treatments in managing fatigue, with the goal of improving the overall well-being of patients.

## Materials and methods

An anonymous international retrospective survey hosted on the Qualtrics programme was disseminated by the authors and several international patient support groups via social media (e.g. Facebook, X or Instagram) from April 12 to May 25, 2023 (Qualtrics 2023). Participants aged 16 and over with a clinical or surgical diagnosis of endometriosis were eligible to self-enrol, with no incentives for completion. A participant information sheet was provided at the start of the questionnaire, explaining the study purpose and how data would be stored. Implied consent was considered if the survey was submitted. Qualtrics assigned cookies to respondents, preventing multiple submissions and removed suspicious IP addresses.

The survey included 37 questions spanning nine webpages, presented in a fixed order (online survey S1). Answers could be changed before final submission. Participants were notified of missed questions before completion, and non-response options (i.e. not applicable) were available. Adaptive questioning was used to ensure only relevant questions were asked (e.g. only participants who used hormonal therapies were asked to rate effectiveness of hormonal therapies). The questionnaire included demographic details and the validated brief fatigue inventory (BFI) (Mendoza *et al.* 1999). The BFI is a simple instrument initially designed for cancer patients. It consists of nine items, each measured on a 0–10 numeric rating scale (0 describing no fatigue/interference and 10 describing fatigue ‘as bad as you can imagine’/‘completely interferes’), correlating with patient symptoms and function. The scores can be further categorised as mild (1–3), moderate (4–6) and severe (7–10) by averaging the responses for all items (Mendoza *et al.* 1999). Changes in fatigue symptoms from various endometriosis treatments over the past 5 years were measured using the patient’s global impression of change scale, and scored as ‘very much worse’ (−3), ‘much worse’ (−2), ‘minimally worse’ (−1), ‘no change’ (0), ‘minimally improved’ (1), ‘much improved’ (2) or ‘very much improved’ (3) (Hurst & Bolton 2004). Questions were reviewed by a research nurse and patient representatives. Ethical approval was obtained from the Edinburgh Medical School Research Ethics Committee (22-EMREC-050). Data were analysed using R version 4.3.2 and packages tidyverse v2.0.0, viridis v0.6.4 and flextable v0.9.4 (R Core Team 2021, Garnier 2023, Gohel 2023, Wickham 2023). Ordinal data are presented as medians and interquartile ranges (IQR). For categorical data, absolute and relative frequencies are given. A correlation analysis between BFI and pelvic pain numerical rating scores (NRS) was performed using Kendall’s tau. A *P*-value <0.05 was considered statistically significant.

## Results

### Recruitment and demographics

In total, 5,632 people accessed the survey and we included 2,907 respondents in our analysis. A total of 2,725 responses were excluded, 391 for suspicious IP addresses and 2,234 for incomplete surveys, making an overall completion rate of 55%. Over 12 countries were represented, mostly from the United Kingdom (58%), Ireland (9%) and Australia (6%) (Supplementary Table 1 (see section on Supplementary materials given at the end of the article)). Surgery was the most common method of diagnosis (71%) followed by imaging (17%) and clinical (12%). Full demographic details and commonly reported comorbidities are summarised in Table 1.

**Table 1:** Demographic data. Data are presented as *n* (%).

Demographic details	Values
Total *n*	2,907
Age	32 (27–38)*
Ethnicity	
Arab	10 (0.3%)
Asian	96 (3.3%)
Black	25 (0.86%)
Hispanic/Latin	146 (5.0%)
Mixed	95 (3.3%)
White	2,523 (86.8%)
Employment	
Prefer not to say	12 (0.4%)
Fully employed	1,828 (62.9%)
Part-time employed	475 (16.3%)
Homemaker	112 (3.9%)
Carer	17 (0.6%)
Student	243 (8.4%)
Unemployed	232 (8.0%)
Endometriosis diagnosis	
Surgical	2,058 (70.8%)
Imaging	488 (16.9%)
Clinical diagnosis	361 (12.4%)
Endometriosis subtype	
Ovarian endometrioma(s)	1,163 (40.0%)
Deep	1,100 (37.8%)
Superficial peritoneal	1,031 (35.5%)
Extra-pelvic	312 (10.7%)
Unknown	812 (27.9%)
Brief fatigue inventory score	
Mild (1–3)	355 (12.2%)
Moderate (4–6)	2,271 (78.1%)
Severe (7–10)	281 (9.7%)
Sleeping time	
<6 h	705 (24%)
6–8 h	1,632 (56%)
>8 h	570 (20%)
Common comorbidities	
Anxiety	1,402 (48.2%)
Depression	955 (32.9%)
Migraine	927 (31.9%)
Irritable bowel syndrome	835 (28.7%)
Adenomyosis	649 (22.3%)
Anaemia	588 (20.2%)

* Data presented as median (IQR).

### Baseline endometriosis and fatigue symptoms

The median endometriosis NRS pain score over the past month was 6 (IQR 5–7). The BFI scores for overall fatigue amongst respondents were moderate (78%), followed by mild (12%) and severe (10%). Correlation analysis of BFI and pelvic pain NRS score showed moderate positive correlation (Kendall’s tau = 0.34, *P* < 0.05). There were 2,100 (72%) participants who were pre-menopausal. Fatigue was reported to be much worsened during menstruation (median: −2, IQR: −3 to −1) and worsened somewhat during ovulation (median: −1, IQR: −2 to 0); both distributions skewed right. The remainder post-menopausal participants reported fatigue symptoms largely unchanged after menopause, with a normal distribution of responses (IQR: −2 to 1). Only 18% (77/422) respondents who had undergone spontaneous/surgical-induced menopause had used hormonal replacement therapy in the past for endometriosis, which did not affect fatigue symptoms.

### Endometriosis treatments and fatigue

**Figure 1:**
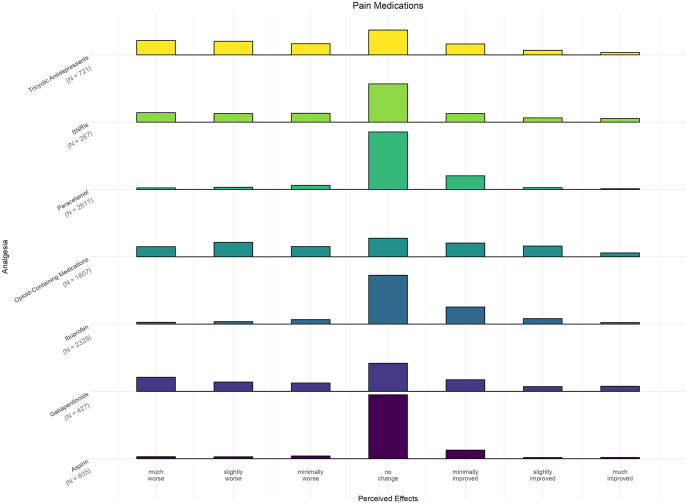
Effect of endometriosis pain medications on fatigue. The bars represent the frequency of responses for each analgesic (e.g. ibuprofen) in endometriosis patients, based on the patient’s global impression of change (PGIC) scale, reflecting perceived effects on fatigue, ranging from ‘much worse’ to ‘much improved’. SNRI, serotonin-norepinephrine reuptake inhibitor.

The most common analgesia used were paracetamol (90%; *n* = 2,611) and ibuprofen (80%; *n* = 2,329); median responses were 0 (IQR: 0–0) and 0 (IQR: 0–1) for changes in fatigue, respectively. When comparing the distribution of responses, simple analgesics (paracetamol, non-steroidal anti-inflammatories and Celebrex) had a majority of responses as ‘no change’. Medications such as tricyclic antidepressants (IQR: −2 to 0), gabapentinoids (IQR: −2 to 1), and opioid-containing analgesia (IQR: −2 to 1) also had median responses for ‘no change’ in fatigue but with a wider distribution of responses to ‘worse/much worse’ (Fig. 1).

**Figure 2:**
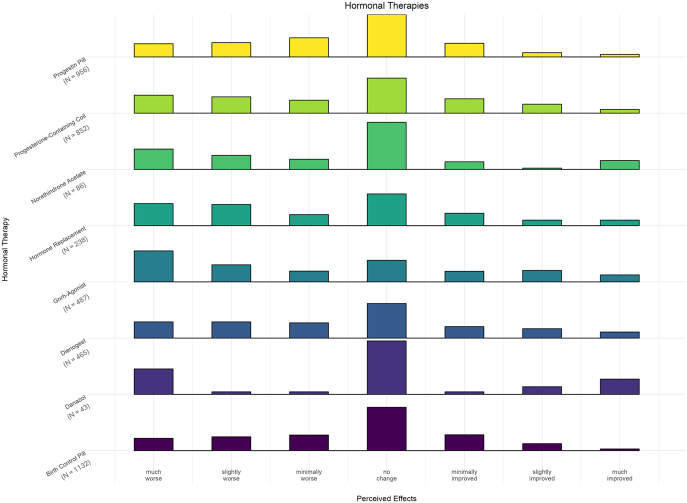
Effect of endometriosis hormonal medications on fatigue. The bars represent the frequency of responses for each hormonal medication (e.g. dienogest) in endometriosis patients, based on the patient’s global impression of change (PGIC) scale, reflecting perceived effects on fatigue, ranging from ‘much worse’ to ‘much improved’.

The most common hormonal management strategies were the combined birth control pills (39%; *n* = 1,132), progesterone-only contraceptive pills (33%; *n* = 956) and progesterone-containing intrauterine devices (29%; *n* = 852) (Fig. 2). Median responses were ‘unchanged’ fatigue responses for all three and IQRs were −1 to 0, −2 to 0 and −2 to 0. Other progesterone medications used for endometriosis such as dienogest (median: 0, IQR: −2 to 0), norethindrone acetate (median: 0, IQR: −2 to 0) and danazol (median: 0, IQR: −2 to 0) had similar ‘unchanged’ fatigue responses. GnRH agonists were previously used by 17% of respondents. It was the only hormonal treatment to have a median response of ‘minimally worse’ fatigue and the largest IQR (−3 to 1). There is a bimodal distribution of results at ‘no change’ (20%; *n* = 96) and ‘very much worse’ (28%; *n* = 138).

**Figure 3:**
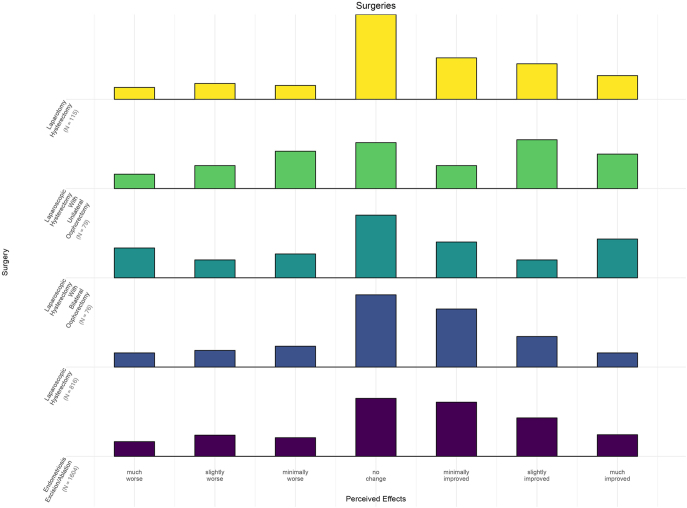
Effect of endometriosis surgeries on fatigue. The bars represent the frequency of responses for different surgeries (e.g. laparoscopic hysterectomy) in endometriosis patients, based on the patient’s global impression of change (PGIC) scale, reflecting perceived effects on fatigue, ranging from ‘much worse’ to ‘much improved’.

Laparoscopic endometriosis excision/ablation was the most common surgical treatment undergone by 55% of respondents (*n* = 1,604) (Fig. 3). The median response was ‘minimally improved’ fatigue (IQR: 0 to 2) with a left skew. Laparotomy excision/ablation of endometriosis was less common (7% of respondents). The median response was ‘unchanged’ fatigue although the distribution of results was similar to laparoscopic surgery. Laparoscopic hysterectomy was the second most common surgery (28%; *n* = 816). Median response was ‘unchanged’ fatigue and IQR of 0 to 1. However, distribution of results was skewed left. Open and transvaginal hysterectomies were less common surgical approaches but also had the same median response of ‘unchanged’ fatigue and distribution of results skewed left. Hysterectomies with bilateral oophorectomy had a median response of ‘no change’ in fatigue following a normal distribution. Laparoscopic/open hysterectomies with unilateral oophorectomy also had a median response of ‘no change’ in fatigue but responses were skewed left (IQRs: −1 to 2).

**Figure 4:**
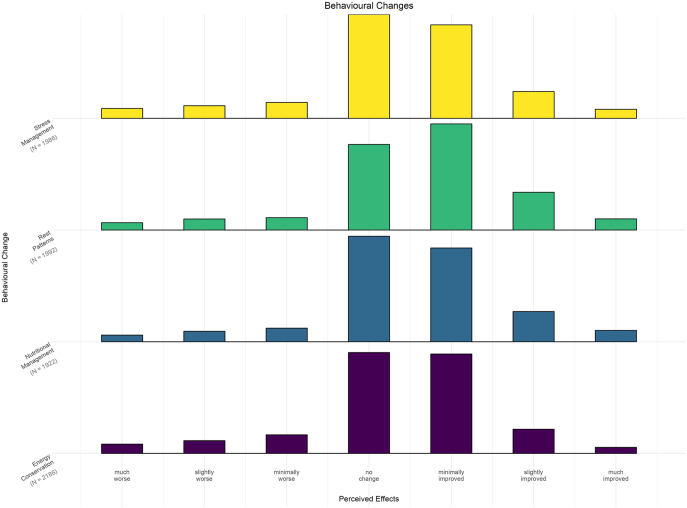
Effect of endometriosis behavioural changes on fatigue. The bars represent the frequency of responses for different behavioural changes (e.g. stress management) in endometriosis patients, based on the patient’s global impression of change (PGIC) scale, reflecting perceived effects on fatigue, ranging from ‘much worse’ to ‘much improved’.

All four behaviour changes were commonly implemented by patients: energy conservation (75%; *n* = 2,186), nutritional management (66%; *n* = 1,922), stress management (68%; *n* = 1,986) and changes in activity/rest patterns (69%; *n* = 1,992) (Fig. 4). Median responses were ‘no change’ in fatigue for all strategies except changes in activity/rest patterns (median = ‘minimal change’). However, responses were distributed similarly across all four strategies between ‘no change’ or ‘minimal improvement’ in fatigue symptoms (IQRs: 0 – 1).

## Discussion

Fatigue continues to be very prevalent in the endometriosis population and a majority of our respondents experienced a moderate severity similar to previous studies (Surrey *et al.* 2019, DiBenedetti *et al.* 2020). The moderate correlation between endometriosis pelvic pain severity and fatigue also supports current evidence (Facchin *et al.* 2021, Soliman *et al.* 2021). Worsened fatigue around the time of menses is unsurprising as dysmenorrhoea may contribute to physical and mental symptoms. Commonly, endometriosis pain symptoms improve during the post-menopausal period due to reduced oestrogen production and subsequent disease progression. However, the unchanged fatigue symptoms after menopause may have been confounded by symptoms of menopause itself or a rarer subset of post-menopausal endometriosis presentation (Vallée *et al.* 2024).

Although endometriosis-associated fatigue may correlate with pelvic pain, it is equally important to recognise the multifactorial nature of fatigue. This survey demonstrated that a wide variety of medical and surgical strategies traditionally targeted for pain often have little to no effect on fatigue. Common first-line medications for pain symptoms such as ibuprofen and combined oral contraceptive pills generally did not improve fatigue symptoms. In fact, patients reported worsened fatigue after pain medications including opioid-containing analgesia (43%) and neuropathic pain medication (39–48%), which could be attributed to their side-effect profiles (Zlott & Byrne 2010, Mu *et al.* 2017).

Negative side-effects such as fatigue, vasomotor symptoms, bone mineral density loss and decreased libido caused by GnRH agonists alone are well recognised due to pituitary suppression and an induced hypoestrogenic state (Veth *et al.* 2023). Thus, hormonal add-back has been increasingly used to help mitigate these negative effects (Surrey 2023). The most recent Cochrane review on all GnRH analogue therapies for endometriosis only identified two studies comparing adverse effects of fatigue against danazol treatment, which showed no significant differences in risk ratios (Veth *et al.* 2023). A trial by Surrey *et al.* found significantly improved endometriosis-associated fatigue with GnRH antagonists and add-back hormone replacement therapy compared to placebo in patients with moderate to severe endometriosis-associated pain. Overall, there is limited evidence directly assessing endometriosis-associated fatigue and GnRH analogue. Whether hormonal add-back was used and GnRH-antagonists were not assessed in our survey, which can be further assessed in future studies.

As mentioned above, fatigue can correlate to pelvic pain. Although median responses to most surgeries showed ‘no change’ in fatigue, a greater proportion of people experienced improvements in symptoms compared to other treatments (especially endometriosis excision/ablation; median score ‘minimally improved’, >50% of responses describing improved fatigue symptoms). These findings support a recent pilot study, which found significantly decreased fatigue at 6 months’ follow-up after surgical removal of endometriosis lesions (Reischer *et al.* 2024). Since our survey did not capture the specific timeframe post-operatively to assess fatigue scores, this may explain our wider range of fatigue improvement levels. Equally, whether the ‘minimal improvement’ translates to clinical significance is beyond the scope of our study and a larger study is needed.

Unsurprisingly, a majority of respondents had tried behavioural modifications for endometriosis management. Unfortunately, most respondents reported ‘no change’ or ‘minimal improvement’ with behavioural modifications, safe and accessible alternative management options for fatigue, which was disappointing. There has been increasing effort from patient support groups and clinicians to encourage self-management strategies, especially following the COVID-19 pandemic (Leonardi *et al.* 2020). Although the improvement in fatigue with rest pattern changes was small, this may be attributed to improvement in sleep. A recent systematic review by Sumbodo *et al.* found a positive relationship between endometriosis pain symptoms, sleep disturbances and quality of life (Sumbodo *et al.* 2024). These conclusions were based on a limited number of publications, but the role of sleep interventions on endometriosis-associated fatigue may be a promising area of future research. Anxiety and depression were also the most common comorbidities reported by participants in our survey and have been strongly associated with greater fatigue and poorer quality of life (Szypłowska *et al.* 2023). A small number of pilot studies have also compared various psychological and mind-body interventions for endometriosis, demonstrating decreased pain and fatigue symptoms although larger sample sizes and higher-quality study design are warranted (Evans *et al.* 2019). Dietary changes such as a low FODMAP diet, decreased meat intake and antioxidants have also been an increasingly popular management strategy within endometriosis research, although outcomes have been pain-focused (Barnard *et al.* 2023). Our survey did not ask for the specific type of dietary changes, as this would be beyond our scope. With chronic inflammatory conditions such as endometriosis, prolonged exposure to pro-inflammatory cytokines may modulate neuronal processes contributing to mental and physical fatigue (Karshikoff *et al.* 2017). However, the role of anti-inflammatory diets on fatigue associated with other chronic diseases has been equivocal, similar to findings from our survey (Haβ *et al.* 2019). Conservative endometriosis therapies remain difficult to research due to heterogeneity. Future large-scale clinical trials are warranted to better understand the impact of these strategies on fatigue.

### Strengths and limitations

To our knowledge, this is the first international survey to compare fatigue symptoms with various endometriosis treatment modalities. Strengths include the large sample size, patient representative involvement during study/questionnaire development and international patient recruitment. Equally, we recognise important limitations of our study. With retrospective survey design, there is risk for recall and temporality bias as participants are asked to draw information from their past experiences. Survey recruitment also relied on self-reported diagnoses, which always has a risk for misclassification or self-selection bias. Assessing fatigue in response to endometriosis treatment is difficult as other comorbidities (e.g. myofascial pain syndrome), psychosocial factors and use of other treatments (e.g. sleep medication or stimulants) may confound results. Participants may have used endometriosis treatment strategies for different durations or multiple concurrently, which can make distinguishing the effect of each modality on fatigue more difficult. We classified the type of endometriosis surgery patients received into broader categories respondents could interpret but procedure intricacies including extent of lesions removed or surgical complexity was not captured, which could influence post-operative fatigue. Other limitations include survey accessibility only to those with internet, self-enrolment, the questionnaire in English with mostly White participants limiting generalisability of results and combining ‘ablation’ and ‘excision’ surgery.

## Conclusion

Endometriosis-related fatigue can have significantly negative impacts on a patient’s quality of life. Overall, our study broadly highlights the ongoing challenges managing endometriosis-related fatigue as majority of the common medical, surgical and behavioural strategies were reported to have no effect on fatigue symptoms. Although some medications may improve pain symptoms, clinicians should be mindful of potential fatigue adverse effects and balance the risks versus benefits in improving overall quality of life. This survey also carries limitations and further research is needed to compare the effects of individual treatment strategies in more detail. Personalised management cannot simply be pain-focused and should consider patient needs holistically.

## Supplementary materials



## Declaration of interest

LHRW has received grant funding from the NIHR and Roche Diagnostics and honorarium (paid to institution) from Gedeon Richter. AH has received payment for a presentation from Theramex. AH is co-editor in chief of *Reproduction and Fertility* but was not involved in the review or editorial process associated with this paper. KKWK, CB, JH and FH have no conflict of interest to declare.

## Funding

AH’s institution (University of Edinburgh) has received payment for consultancy and grant funding from Roche Diagnostics to assist in the early development of a blood diagnostic biomarker for endometriosis. AH’s institution has received payment for consultancy fees from Roche Diagnostics, Gesynta and Joii. 

## Author contribution statement

KKWK, CB and AH were responsible for conception and designing the study. The study was conducted and analysed by KKWK and CB. The data were interpreted by FH, LHRW and JH. The manuscript was written by KKWK and all authors provided critical content editing.
