# Supplementary material for: Perceived effectiveness of endometriosis therapies on fatigue: an international survey
Source: Reprod Fertil. 2025 May 15;6(2):e250010. doi: 10.1530/RAF-25-0010 (PMC12084798; doi:10.1530/RAF-25-0010)
Supplement: Supplementary file 1 [file supplementary_materials.pdf]

Supplementary Table 1. Responses by country.

**Country**	**Number (%)**
Australia	175 (6.02%)
Austria	46 (1.58%)
Belgium	41 (1.41%)
Canada	115 (3.96%)
Germany	17 (0.58%)
Ireland	270 (9.29%)
Italy	49 (1.69%)
Mexico	104 (3.58%)
Netherlands	83 (2.86%)
Norway	49 (1.69%)
United Kingdom of Great Britain and Northern Ireland	1698 (58.41%)
United States of America	113 (3.89%)
Other	147 (5.06%)

Introduction: Thank you for considering participating in our survey. This questionnaire is intended to be entirely anonymous so please avoid entering you name in free text boxes. You are free to withdraw at any point by exiting this page without clicking the final 'submit'.

1. How would you describe your gender? (Tick box)
   - Female
   - Male
   - Non-binary
   - Transgender
   - Other
   - Prefer not to say

2. What is your age? (drop down)

3. Which of these best describes your ethnicity (drop down)?
   - Asian
   - Black
   - Caucasian
   - Hispanic/Latin American
   - Mixed race
   - South Asian or Indian
   - Other:_________
   - Prefer not to say

4. Which country do you live in? (drop down)

5. Please describe your current employment status: (tick box)

   - Fully employed; includes self-employment
   - Part-time employed
   - Unemployed
   - Homemaker
   - Carer
   - Student

6. Height [feet/cm] and weight [kg/lbs/stone]

[open answer questions]

7. Please describe how your endometriosis was diagnosed (tick box):
   - Confirmed by surgery (with the goal of investigating endometriosis)
   - Diagnosed incidentally during surgery (operation for something else and the surgeon found endometriosis)
   - Confirmed by imaging (ultrasound/MRI) with the goal of investigating endometriosis
   - Diagnosed incidentally by imaging (ultrasound/MRI) (incidental findings from a scan for something else)
   - Symptoms suggestive of endometriosis, but diagnosis not yet confirmed

8. Which type of endometriosis do you have? [select all that apply] (tick box)
   - Superficial peritoneal; includes bowels and bladder
   - (Ovarian) endometrioma
   - Deep (infiltrating); includes bowels and bladder
   - Outside the pelvis (e.g. lungs, belly button)
   - Don't know

9. Have you had pelvic **pain in the last month**? (tick box)
   - Yes (go to question 10)
   - No (go to question 11)

10. How would you rate your daily pelvic/abdominal **pain** on average over the past month?

    Average pelvic pain over last 4 weeks?

0	1	2	3	4	5	6	7	8	9	10

No pain　　Moderate pain　　Worst possible

Brief Fatigue Inventory

11. Please rate your **fatigue** (weariness, tiredness) by circling the one number that best describes your fatigue right NOW. (0 to 10; 0 no fatigue – 10 as bad as you can imagine)

12. Please rate your **fatigue** (weariness, tiredness) by circling the one number that best describes your USUAL level of fatigue during past 24 hours. (0 to 10; 0 no fatigue – 10 as bad as you can imagine)

13. Please rate your **fatigue** (weariness, tiredness) by circling the one number that best describes your WORST level of fatigue during past 24 hours. (0 to 10; 0 no fatigue – 10 as bad as you can imagine)

14. Circle the one number that describes how, during the past 24 hours, **fatigue** has interfered with your:
    1. General activity (0 to 10; does not interfere – completely interferes)
    2. Mood (0 to 10; does not interfere – completely interferes)
    3. Walking ability (0 to 10; does not interfere – completely interferes)
    4. Normal work (includes both work outside the home and daily chores) (0 to 10; does not interfere – completely interferes)
    5. Relations with other people (0 to 10; does not interfere – completely interferes)
    6. Enjoyment of life (0 to 10; does not interfere – completely interferes)

15. Have you gone through menopause (menopause with age = 12 months without a period; medical = medication-induced menopause; surgical = ovaries removed)?

- Yes (go to question 17)
- No (go to question 16)

16. Compared to other times during your menstrual cycle, do you notice if menstruation or ovulation affects **fatigue**? (rated very much improved [1], much improved [2], minimally improved [3], no change [4], minimally worse [5], much worse [6], or very much worse [7])
    a. Menstruation
    b. Ovulation

17. Have you noticed any changes in **fatigue** symptoms after menopause (rated very much improved [1], much improved [2], minimally improved [3], no change [4], minimally worse [5], much worse [6], or very much worse [7])

Current Treatments

Sleep Questions

18. Please describe your average sleeping time (hours) per 24 hours: (tick box)
    - Less than 6 hours
    - 6 to 8 hours
    - More than 8 hours

19. Do you currently take any kind of medication/treatment or make any lifestyle changes to help go to sleep? (tick box)
    Yes (go to question 20)
    No (go to question 21)

20. Please specify which kind of sleep medication/treatment/lifestyle modifications you take? (tick box)
    - Non-benzodiazepine hypnotic medication ‘Z’-drugs (e.g. zopiclone, zaleplon, zolpidem)
    - Sedating antihistamine (e.g. promethazine)
    - Melatonin receptor agonists (e.g. Ramelteon)
    - Orexin receptor antagonists (e.g. Suvorexant)
    - Tricyclic antidepressant (e.g. doxepin, amitriptyline)
    - Serotonin modulator (e.g. trazodone)
    - Benzodiazepine (e.g. temazepam)
    - Lifestyle changes (e.g. sleep hygiene, avoiding electronic screens before bed, avoiding caffeine in the evening, etc…)
    - Cognitive behavioural therapy for insomnia
    - Others: _______

21. Have you tried stimulants (eg. …) for **fatigue** <u>in the past 5 years</u>?
    Yes (go to question 22)
    No (go to question 23)

22. When you were taking stimulant(s), did you notice a change in your fatigue symptoms? [select all that apply] (rated very much improved [1], much improved [2], minimally improved [3], no change [4], minimally worse [5], much worse [6], or very much worse [7])
    - Caffeine (e.g. coffee, tea, cola)
    - Amphetamines (e.g. Adderall, Dexedrine)
    - Methylphenidate (e.g. Ritalin, Concerta)
    - Ephedrine (e.g. spray/supplement)
    - Pseudoephedrine (e.g. spray/supplement)
    - Modafinil (e.g. Modasomil)
    - Other:________

Hormonal Therapy Questions

23. Have you used hormonal therapy for endometriosis symptoms <u>in the past 5 years</u>? (tick box)
    Yes (go to question 24)
    No (go to question 26)

24. Did you notice a change in **<u>fatigue</u>** symptoms while on hormonal therapy? (tick box)

    Worsened (go to question 25)
    Better (go to question 25)
    Unchanged (go to question 25)
    Don't know (go to question 26)

25. When you were taking any hormonal therapies listed below, did you notice a change in your **<u>fatigue</u>** symptoms? (rated very much improved [1], much improved [2], minimally improved [3], no change [4], minimally worse [5], much worse [6], or very much worse [7])

- Combined birth control pill (e.g. Marvelon, Yasmin, Microgynon)
- Progestin only birth control pill ("mini-pill", e.g. Cerazette, Micronor)
- Unsure of which type of oral birth control pill
- Progestin injection/shot (e.g. Depoprovera)
- Transdermals: patches (e.g. OrthoEvra, Climara), dots (Vivelle dot)
- Vaginal ring (NuvaRing)
- Progesterone containing coil/IUD (Mirena, Kyleena, Jaydess)
- Hormonal implant (Implanon/Nexplanon)
- Oral progestins to regulate the cycle (e.g. medroxyprogesterone acetate [Provera], dydrogesterone [Duphaston], dienogest [Visanne], Norethisterone)
- GnRH agonist injection/shot (e.g. decapeptyl, leuprolilide (leuproline) acetate [Prostap or Lupron], goserelin [Zoladex])
- Norethindrone acetate (Aygestin)
- Danazol
- Hormone replacement therapy (e.g. Premarin, Provera)
- Other (please specify):___________
- Don't know what type of hormone

Pain Medicine Questions

26. Have you used pain medication for endometriosis symptoms in the past 5 years? (tick box)
    Yes (go to question 27)
    No (go to question 29)

27. When you were taking pain medicine, did you notice a change in your **fatigue** symptoms? (tick box)

    Worsened (go to question 28)
    Better (go to question 28)
    Unchanged (go to question 28)
    Don't know (go to question 28)

28. When you were taking the pain medication(s) listed below, did you notice a change in your **fatigue** symptoms? (rated very much improved [1], much improved [2], minimally improved [3], no change [4], minimally worse [5], much worse [6], or very much worse [7])

- Paracetamol/acetaminophen
- Aspirin (325 mg or more/tablet)
- Ibuprofen (e.g., Brufen)
- Celebrex, Vioxx (COX-2 inhibitors)
- Other anti-inflammatory analgesics (naproxen, mefanamic acid, Aleve, Naprosyn, Relafen, Ketoprofen, Anaprox)
- Stronger analgesics (hydrocodone + paracetamol, codeine+paracetamol, morphine, codeine, oxycodone, hydrocodone, Demerol)
- Tricyclic antidepressants (e.g. amitriptyline, nortriptyline, imipramine)
- Gabapentinoids (e.g. gabapentin, pregabalin)
- SNRIs (e.g. duloxetine,milnacipran)
- Muscle relaxants (diazepam/temazepam, buscopan)
- Other (please specify):___________

Surgery Questions

29. Have you had surgery for endometriosis symptoms?
    Yes (go to question 30)
    No (go to question 32)

30. Did you notice a change in **fatigue** symptoms after your most recent surgery?
    Worsened (go to question 31)
    Better (go to question 31)
    Unchanged (go to question 31)
    Don't know (go to question 32)

31. After your most recent surgery, did you notice a change in your **fatigue** symptoms? (rated very much improved [1], much improved [2], minimally improved [3], no change [4], minimally worse [5], much worse [6], or very much worse [7])

- Laparoscopic (keyhole surgery) Hysterectomy without having ovaries removed
- Laparotomy (open surgery) Hysterectomy without having ovaries removed
- Transvaginal Hysterectomy without having ovaries removed

- Laparoscopic (keyhole surgery) Hysterectomy with having both ovaries removed
- Laparotomy (open surgery) Hysterectomy with having both ovaries removed
- Transvaginal Hysterectomy with having both ovaries removed
- Laparoscopic (keyhole surgery) Hysterectomy with having only one ovary removed
- Laparotomy (open surgery) Hysterectomy with having only one ovary removed
- Transvaginal Hysterectomy with having only one ovary removed
- Laparoscopic (keyhole surgery) endometriosis excision or ablation
- Laparotomy (open surgery) endometriosis excision or ablation
- Diagnostic laparoscopy (keyhole surgery)
- Laparotomy (open surgery) but unsure about details
- Laparoscopy (keyhole surgery) but unsure about details
- Other abdominal surgery: ....

Behaviour Change Questions

32. Have you tried behavioural changes (eg strategies for energy conservation, nutrition, stress management,…) for **fatigue** in the past 5 years?
    Yes (go to question 33)
    No (go to question 35)

33. Did you notice changes in your **fatigue** symptoms after behavioural changes (eg strategies for energy conservation, nutrition, stress management,…)?
    Worsened (go to question 34)
    Better (go to question 34)
    Unchanged (go to question 34)
    don’t know (go to question 35)

34. Did you notice changes in your **fatigue** symptoms after the making behavioral changes? Please choose the ones you used and rate them. (rated very much improved [1], much improved [2], minimally improved [3], no change [4], minimally worse [5], much worse [6], or very much worse [7])

    - Energy conservation strategies (for example require assistance for household activities, taking breaks throughout the day)

    - Nutritional management strategies (for example small meals throughout the day, meal-prep, work with dietitian, intake of complex carbohydrates)

    - Stress management strategies (for example identify fatigue triggers, distraction techniques, engage in social support/support groups)

    - Strategies to regulate daily activity and rest patterns (Planning around an activity diary, several short naps throughout the day, lighter exercises, only doing activities you enjoy)

Current Treatments for Fatigue

35. What **current** strategies/treatments do you use to manage your **fatigue**? (tick all that apply)

None

Sleep medication

- Non-benzodiazepine hypnotic medication 'Z'-drugs (e.g. zopiclone, zaleplon, zolpidem)
- Sedating antihistamine (e.g. promethazine)
- Melatonin receptor agonists (e.g. Ramelteon)
- Orexin receptor antagonists (e.g. Suvorexant)
- Tricyclic antidepressant (e.g. doxepin, amitriptyline)
- Serotonin modulator (e.g. trazodone)
- Benzodiazepine (e.g. temazepam)
- Lifestyle changes (e.g. sleep hygiene, avoiding electronic screens before bed, avoiding caffeine in the evening, etc…)
- Cognitive behavioural therapy for insomnia
- Others: _______

Stimulants

- Caffeine (e.g. coffee, tea, cola)
- Amphetamines (e.g. Adderall, Dexedrine)
- Methylphenidate (e.g. Ritalin, Concerta)
- Ephedrine (e.g. spray/supplement)
- Pseudoephedrine (e.g. spray/supplement)
- Modafinil (e.g. Modasomil)
- Other:________

Hormone therapy

- Combined birth control pill (e.g. Marvelon, Yasmin, Microgynon)
- Progestin only birth control pill ("mini-pill", e.g. Cerazette, Micronor)
- Unsure of which type of oral birth control pill
- Progestin injection/shot (e.g. Depoprovera)
- Transdermals: patches (e.g. OrthoEvra, Climara), dots (Vivelle dot)
- Vaginal ring (NuvaRing)
- Progesterone containing coil/IUD (Mirena, Kyleena, Jaydess)
- Hormonal implant (Implanon/Nexplanon)
- Oral progestins to regulate the cycle (e.g. medroxyprogesterone acetate [Provera], dydrogesterone [Duphaston], dienogest [Visanne], Norethisterone)
- GnRH agonist injection/shot (e.g. decapeptyl, leuprolilide (leuproline) acetate [Prostap or Lupron], goserelin [Zoladex])
- Norethindrone acetate (Aygestin)
- Danazol
- Hormone replacement therapy (e.g. Premarin, Provera)
- Other (please specify):___________

Pain medicine

- Paracetamol/acetaminophen
- Aspirin (325 mg or more/tablet)
- Ibuprofen (e.g., Brufen)
- Celebrex, Vioxx (COX-2 inhibitors)
- Other anti-inflammatory analgesics (naproxen, mefanamic acid, Aleve, Naprosyn, Relafen, Ketoprofen, Anaprox)

Stronger analgesics (hydrocodone + paracetamol, codeine+paracetamol, morphine, codeine, oxycodone, hydrocodone, Demerol)
Tricyclic antidepressants (e.g. amitriptyline, nortriptyline, imipramine)
Gabapentinoids (e.g. gabapentin, pregabalin)
SNRIs (e.g. duloxetine,milnacipran)
Muscle relaxants (diazepam/temazepam, buscopan)
Other (please specify):___________

Behavioral techniques

Energy conservation strategies (require assistance for household activities, taking breaks throughout the day)
Nutritional management strategies (small meals throughout the day, meal-prep, work with dietitian, intake of complex carbohydrates)
Stress management strategies (identify fatigue triggers, distraction techniques, engage in social support/support groups)
Strategies to regulate daily activity and rest patterns (Planning around an activity diary, several short naps throughout the day, lighter exercises, only doing activities you enjoy)

Other:_________

Other Medical Conditions

36. Do you have any other conditions that may contribute to fatigue? [select all that apply] (tick box)

Anaemia
Pernicious anaemia (B12 deficiency)
Anxiety
Chronic fatigue syndrome
Fibromyalgia
Heavy menstrual bleeding
Hypothyroidism
Long covid
Depression
Adenomyosis
Other:__________

37. Do you currently have/previously had any of the following medical conditions? [select all that apply] (tick box)

Anxiety requiring medication or therapy
Asthma
Cardiovascular disease
Crohn’s Disease
Deafness/difficulty hearing
Depression requiring medication or therapy
Diabetes requiring diet control
Diabetes requiring insulin or tablets
Eczema
Fibroids
Glandular Fever
Graves’ Disease

Hashimoto's disease
High blood pressure
Irritable Bowel Syndrome (IBS)
Migraine
Mitral valve prolapse
Multiple Sclerosis
Painful bladder/interstitial cystitis (NOT bacterial bladder infection)
Pelvic Inflammatory Disease (PID)
Polycystic Ovary Syndrome
Rheumatoid Arthritis
Scoliosis (curvature of the spine)
Spine problems (excluding scoliosis)
Sjogren's syndrome
Sleep apnoea syndrome
SLE (Lupus)
Thyroid disease
Ulcerative Colitis
Other (*Please specify*):

38. Do you have any further comments you would like to make about your experience of fatigue and measures that might have had an influence on that symptom? Responses to this question will not be used in our data analysis but for planning future surveys on fatigue. Please do not enter any details that could identify you or another individual. (maximum 100 words)

Closing: This is the end of the survey. We would like to thank you again for your time and participation. If you are happy to submit your anonymous responses, please click the final 'submit' button.
